# Proteomic Analysis of the Periodontal Ligament During Orthodontic Movement: A Study in Rats

**DOI:** 10.3390/proteomes13030042

**Published:** 2025-09-11

**Authors:** Camila Chierici Marcantonio, Maria Eduarda Scordamaia Lopes, Lélio Fernando Ferreira Soares, Cristiane Ribeiro Salmon, Francisco Humberto Nociti Junior, James Deschner, Andressa Vilas Boas Nogueira, Joni Augusto Cirelli

**Affiliations:** 1 Department of Diagnosis and Surgery, School of Dentistry at Araraquara, Sao Paulo State University—UNESP, Araraquara 14801903, São Paulo, Brazil; camila.marcantonio@unesp.br (C.C.M.); maria.e.lopes@unesp.br (M.E.S.L.); lelio.soares@unesp.br (L.F.F.S.); 2 Department of Prosthodontics and Periodontics, Division of Periodontics, Piracicaba Dental School, University of Campinas—UNICAMP, Piracicaba 13414903, São Paulo, Brazil; cris.salmon.bb@gmail.com; 3 Department of Comprehensive Dentistry, University of Maryland School of Dentistry, Baltimore, MD 21201, USA; fhnociti@gmail.com; 4 Department of Periodontology and Operative Dentistry, University Medical Center of the Johannes Gutenberg University, 55131 Mainz, Germany; james.deschner@uni-mainz.de (J.D.); a.nogueira@uni-mainz.de (A.V.B.N.)

**Keywords:** proteomics, orthodontic tooth movement, orthodontics, periodontal ligament, proteomics

## Abstract

The periodontal ligament (PDL) is a dynamic connective tissue that absorbs and transmits mechanical forces, playing a critical role during orthodontic tooth movement (OTM). This study aimed to characterize the proteomic profile of rat PDLs subjected to OTM. Ten Holtzman rats were allocated into Control and OTM groups. After 15 days of force application, hemimaxillae were harvested, and PDL tissues from the first maxillary molars were isolated via laser capture microdissection. Protein extracts were analyzed using liquid chromatography–tandem mass spectrometry (LC-MS/MS), followed by quantitative and enrichment analyses. Immunohistochemistry was performed to validate selected proteins. The full proteomic datasets supporting these findings are available in the PRIDE repository under the identifiers PXD055817 and PXD033647. A total of 1121 proteins were identified; 101 were exclusive to the OTM group, 324 to the control, and 696 shared. Among the 335 proteins with differential abundance, 334 were downregulated and one (Prelp) was upregulated in the OTM group. Enrichment analysis revealed that differentially abundant proteins were associated with molecular functions such as protein binding, and cellular components including extracellular exosomes, focal adhesions, and the extracellular matrix. Immunohistochemical analysis confirmed the presence of Prelp, Rbm3, and Cirbp in PDL tissues. These findings demonstrate that OTM significantly alters the proteomic landscape of the PDL and identify key proteins potentially involved in periodontal remodeling.

## 1. Introduction

Orthodontic tooth movement (OTM) is driven by the application of mechanical forces to the periodontal supporting tissues, including the alveolar bone, periodontal ligament (PDL), and cementum. These forces trigger a cascade of biological responses mediated by fibroblasts and osteoclasts, leading to an aseptic local inflammatory reaction that initiates bone remodeling and subsequent tooth movement [1,2]. This process is accompanied by the release of several pro-inflammatory cytokines, such as interleukin-1 beta (IL-1β), interleukin-6 (IL-6), and tumor necrosis factor-alpha (TNF-α) [3,4,5].

Cells within the PDL and alveolar bone are particularly responsive to mechanical stimuli, facilitating dynamic interactions between genetic and environmental factors. These cells are central to the regulation and progression of orthodontic tooth movement (OTM) [6]. The PDL itself is a specialized connective tissue predominantly composed of fibroblasts [7], which plays critical roles in absorbing and transmitting mechanical loads, as well as supplying nutrients and maintaining vascular support to the surrounding structures [8].

In response to orthodontic forces, cells within the PDL and alveolar bone initiate complex cell-to-cell communication through both direct contact and the release of signaling molecules. This mechanical loading activates several molecular pathways, such as MAPK, NF-κB, and Wnt/β-catenin, which regulate gene expression, protein synthesis, and cytoskeletal remodeling [9,10,11]. Additionally, dynamic protein–protein interactions contribute to extracellular matrix (ECM) reorganization and the modulation of inflammatory responses, enabling a coordinated cellular adaptation essential for effective bone remodeling during OTM [12,13].

Proteomic approaches have emerged as powerful tools to investigate the effects of various stimuli on tissue- and cell-specific protein abundance profiles. These methods offer valuable insights into complex biological mechanisms [14,15,16,17,18]. Prior research has explored the proteomic landscape of human periodontal ligament fibroblasts (hPDLFs) in vitro [19,20,21], and comparative analyses between hPDLFs and human gingival fibroblasts (hGFs) have highlighted functional distinctions reflected in their proteomic signatures [20].

Despite these advances, there remains a notable gap in studies evaluating in vivo PDL proteome alterations in response to mechanical loading. Therefore, the present study aimed to investigate the proteomic profile of the PDL in rats subjected to orthodontic forces, contributing to a deeper understanding of the molecular mechanisms underlying OTM.

## 2. Materials and Methods

### 2.1. Animals

This study was conducted in accordance with the ARRIVE (Animal Research: Reporting of In Vivo Experiments) guidelines and was approved by the Ethical Committee on Animal Experimentation of the São Paulo State University, School of Dentistry at Araraquara, Brazil (protocol number 16/2015).

A total of ten male adult Holtzman rats, with an average weight of approximately 300 g, were used and randomly assigned into two experimental groups (*n* = 5 per group): Control (C), which did not undergo any intervention, and Orthodontic Tooth Movement (OTM), in which the first maxillary molar was selected to receive orthodontic force for a duration of 15 days. All animals were housed under standard laboratory conditions, including a controlled ambient temperature of 22–25 °C, a 12 h light/dark cycle, and free access to water and a standard laboratory diet.

### 2.2. OTM Procedure

General anesthesia was induced via intramuscular injection using ketamine hydrochloride 10% (0.08 mL/100 g body weight) combined with xylazine hydrochloride 2% (0.04 mL/100 g body weight). To apply the orthodontic force, a closed-coil nickel-titanium (NiTi) spring (Sentalloy, GAC Central Slip, New York, NY, USA) was attached between the maxillary first molar and the central incisors using a 0.20 mm stainless steel wire (CrNi, 55.01.208; Morelli, Sorocaba, Brazil). Grooves were created around the incisors to facilitate proper placement, and the apparatus was stabilized with a thin layer of composite resin. On the molar side, the spring was positioned on the occlusal surface of the first molar and similarly secured with composite resin. To eliminate occlusal interferences and ensure unrestricted tooth movement, the mandibular first molars were extracted [22]. The nickel-titanium spring exerted a relatively constant orthodontic force of approximately 25 g throughout the 15-day experimental period.

### 2.3. Laser Capture Microdissection (LCM) and Protein Extraction

Fifteen days after the initiation of OTM, animals were euthanized by anesthetic overdose. Maxillae were carefully harvested and histologically processed to obtain serial sections in the buccolingual direction, as previously described [17]. Briefly, the specimens were fixed in 10% buffered formalin (Fisher Diagnostics, Middletown, VA, USA) at 4 °C for 24 h, followed by three rinses in phosphate-buffered saline (PBS; pH 7.4, Applied Biosystems, Foster City, CA, USA) for 30 min each at 4 °C. Decalcification was performed in 20% ethylenediaminetetraacetic acid (EDTA; Merck/Millipore, Darmstadt, Germany) for 30 days at 4 °C under constant agitation. Following decalcification, samples were trimmed, rinsed in PBS (30 min, 4 °C), dehydrated in 100% ethanol (30 min, 4 °C), cleared in xylene (20 min, room temperature), and embedded in paraffin. Paraffin blocks were stored at −20 °C until sectioning.

Serial 5 μm thick buccolingual sections of the first molar region were obtained using a microtome and mounted on polyethylene naphthalate (PEN) membrane glass slides (Applied Biosystems, Foster City, CA, USA). Sections were deparaffinized in two changes of xylene for 2 min each, followed by a third xylene wash for 5 min, and air-dried for 5 min prior to microdissection. PDL tissues were then microdissected under sterile conditions, as previously described [23]. For each sample, 8 to 10 sections were microdissected, and the total captured area was measured to normalize the amount of collected tissue and protein used for downstream liquid chromatography–tandem mass spectrometry (LC-MS/MS) analysis (LTQ-Orbitrap Velos ETD, Thermo Scientific, Waltham, USA).

Tissues captured on PEN membrane caps were incubated with 30 μL of 8 M urea for 30 min at room temperature to extract proteins. The samples were subsequently sonicated and briefly centrifuged. Total protein extracts were reduced using 5 mM dithiothreitol (DTT), alkylated with 14 mM iodoacetamide, and enzymatically digested with 2 μg of trypsin, following the protocol established by Salmon, Giorgetti, Leme, Domingues, Sallum, Alves, Kolli, Foster, and Nociti Jr [17]. Tryptic peptides were then acidified to pH 2.0 using formic acid, desalted and purified with ZipTip^®^ C18 microcolumns (P10 pipette tips, Merck Millipore, Billerica, MA, USA), dried using a vacuum concentrator, and reconstituted in 0.1% formic acid for subsequent proteomic analysis.

### 2.4. LC-MS/MS and Bioinformatics Analysis

Mass spectrometry analysis was performed at the BIOMASS Core Facility for Scientific Research, University of São Paulo, Brazil. Tryptic peptide mixtures were analyzed using an LTQ-Orbitrap Velos ETD mass spectrometer (Thermo Scientific, Waltham, MA, USA) coupled to an Easy NanoLC II nanoflow liquid chromatography system (Thermo Scientific). Peptide separation was achieved using a linear gradient of 2–95% acetonitrile in 0.1% formic acid over 105 min, at a constant flow rate of 300 nL/min, on a C18 PicoFrit analytical column (C18 PepMap, 75 µm id × 10 cm, 3.5 µm particle size, 100 Å pore size; New Objective, Ringoes, NJ, USA).

The mass spectrometer operated in positive ion mode with data-dependent acquisition. Full MS scans were acquired in the Orbitrap Exploris 480 (Thermo Fisher Scientific, Bremen, Germany) at a resolution of 60,000 FWHM across a 400–1500 m/z range, with an automatic gain control (AGC) target of 1 × 10^6^ and a maximum injection time of 500 ms. The 20 most intense precursor ions from each full scan were selected for fragmentation via collision-induced dissociation (CID) using a normalized collision energy of 35%. Dynamic exclusion was enabled for 30 s to avoid repeated fragmentation of the same ion. Each sample was analyzed in two technical replicates, which were combined for downstream analysis.

Raw data were acquired using Xcalibur software version 2.3 (Thermo Fisher Scientific) and analyzed with Proteome Discoverer (v1.4.0.288, Thermo Finnigan, San Jose, CA, USA). Tandem mass spectra (MS/MS) were searched against the *Rattus norvegicus* UniProt Protein Database (release: 22 January 2014; 51,116 entries), assuming trypsin digestion with up to two missed cleavages. The search parameters included a parent ion mass tolerance of 20 ppm and fragment ion mass tolerance of 0.6 Da. Carbamidomethylation of cysteine (+57 Da) was set as a fixed modification, and oxidation of methionine (+16 Da) was set as a variable modification.

Label-free protein identification and quantification were performed in Proteome Discoverer. Protein abundance was calculated based on normalized spectral counts, and quality filtering was applied prior to differential analysis. Proteins were considered identified if they met the following thresholds: a minimum identification probability of 99% at the protein level (false discovery rate < 1%), and at least one peptide identified with ≥60% probability. XCorr score cutoffs were set as follows: +1 > 1.8, +2 > 2.2, +3 > 2.5, and +4 > 3.5. Normalized spectral count (NSC) values were used to estimate relative protein abundances. Proteins with at least two valid values in at least two samples were retained for differential analysis.

Statistical comparisons between the Control and OTM groups were performed using independent samples t-tests. Protein abundance ratios were calculated based on the average normalized spectral intensities. Principal Component Analysis (PCA) was conducted in R to assess proteomic variability across samples. For unsupervised clustering, normalized spectral count (NSC) values were converted to z-scores and visualized using the EXPANDER software version 4.1. Gene Ontology (GO) enrichment analysis was performed using the DAVID platform (v6.8; https://david.ncifcrf.gov, accessed on 15 January 2019), focusing on Molecular Function (MF) and Cellular Component (CC) categories. Significantly enriched GO terms were identified using a *p*-value threshold of ≤0.05, adjusted for multiple comparisons using the Benjamini–Hochberg correction. Protein–protein interaction (PPI) networks were explored using the STRING database (https://string-db.org, accessed on 15 January 2019), which integrates known and predicted associations to reveal potential functional relationships among differentially abundant proteins (DAPs).

Differential protein abundance was assessed using the beta-binomial statistical test applied to NSC values, with significance defined by a log_2_ fold change > 1.5 and a −log_10_(*p*-value) ≥ 1.

### 2.5. Immunohistochemistry (IHC)

To validate selected proteins identified in the PDL tissues by proteomic analysis, IHC was performed on additional histological sections obtained from the same animals used in LC-MS/MS analysis (*n* = 5 animals per group). IHC was conducted on paraffin-embedded sections using an avidin-biotin complex (ABC) method (Vector Laboratories, Burlingame, CA, USA), with 3-amino-9-ethylcarbazole (AEC; Vector Laboratories) as the chromogenic substrate, as described previously [24]. The primary antibodies used were directed against Prelp (bs13707R, Bioss, Woburn, MA, USA), Cirbp (sc-293325, Santa Cruz Biotechnology, Dallas, TX, USA), and RBM3 (PAC-5-51976, Thermo Fisher, Waltham, MA, USA). Immunostained slides were developed with 3,3′-diaminobenzidine (DAB) and counterstained with Carazzi’s hematoxylin. Negative control sections omitting the primary antibody were included to assess nonspecific background staining.

### 2.6. Statistical Analysis

For IHC analysis, intergroup comparisons were performed using unpaired Student’s *t*-tests (GraphPad Prism 8.0; La Jolla, CA, USA). A *p*-value of <0.05 was considered statistically significant.

## 3. Results

### 3.1. Proteomic Analysis of PDL in the Control and OTM Groups

**Figure 1:**
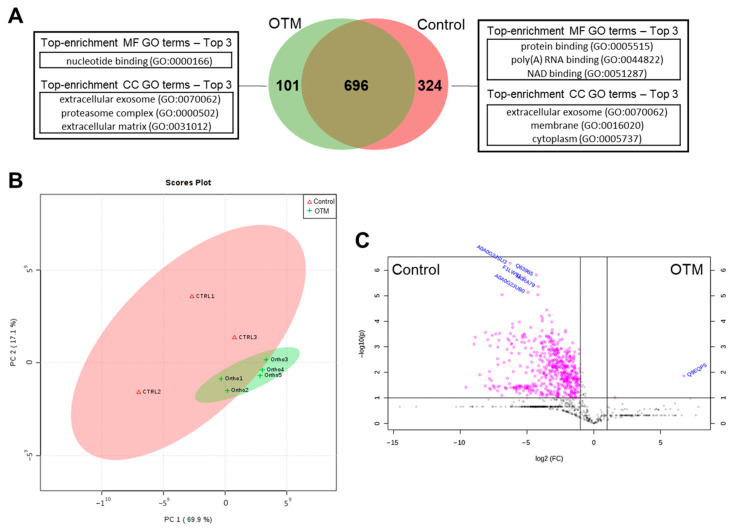
Protein profile summary of OTM and Control groups. (**A**) Venn diagram showing the distribution of the total proteins identified in both groups and the top 3 GO-enrichment terms of MF and CC on each group and between them (*p* < 0.05). (**B**) Principal component analysis graph (PCA) showing the difference between the samples of the Control and OTM groups and the similarity between the samples within each group. (**C**) Volcano plot illustrating DAPs of Control and OTM (log_2_ fold change > 1.5 and a −log_10_(*p*-value) ≥ 1).

Although five animals were assigned to the Control group, only three samples produced proteomic data of sufficient quality for inclusion in the analysis due to technical issues during tissue capture. A total of 1121 proteins were identified, of which 696 (62%) were shared between control- and OTM-derived PDL samples, 324 (29%) were exclusive to the control group, and 101 (9%) were exclusive to the OTM group (Figure 1A). Among these, 335 proteins (30%) showed differential abundance, with 334 significantly downregulated and only one upregulated in the OTM group (*p* < 0.05) (Figure 1C, Table 1). PCA revealed a clear separation between groups, with OTM-derived samples displaying high homogeneity and control samples showing greater variability (PC1 = 69.9%, PC2 = 17.1%) (Figure 1B). The volcano plot highlights proteins exhibiting >1.5-fold change with statistical significance (*p* < 0.05), confirming a strong predominance of downregulated proteins in the OTM group (Figure 1C). A complete list of significantly abundant proteins is available in Supplementary Material Table S1. Raw proteomic data for the Control and OTM groups are available in Supplementary Tables S2 and S3.

**Figure 2:**
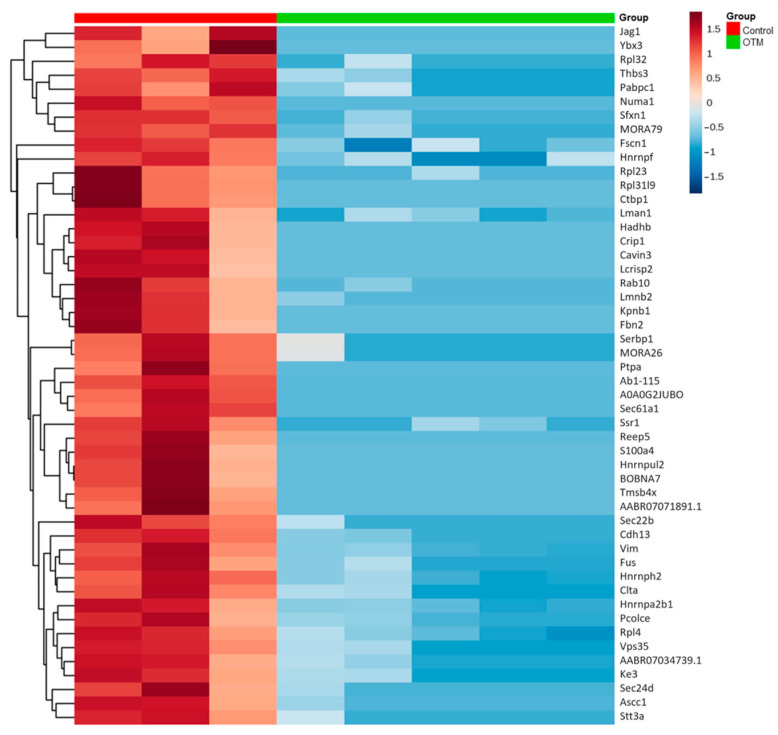
Heat map showing hierarchical clustering of top 50 up- and downregulated proteins from both groups, ranked by *p*-value (*p* < 0.05; ANOVA followed by Tukey’s post hoc test) calculated using the Z-score calculation on log_2_ spectral counts values, applying the Euclidian distance method and average linkage.

A heatmap of the top differentially abundant proteins further illustrates the marked contrast between groups, with clear clustering distinguishing control and OTM samples (Figure 2). Downregulated proteins in the OTM group showed consistently lower abundance across all replicates, while the control group displayed uniformly higher abundance levels.

### 3.2. Biological Characterization of Proteins with Differential Abundance

**Figure 3:**
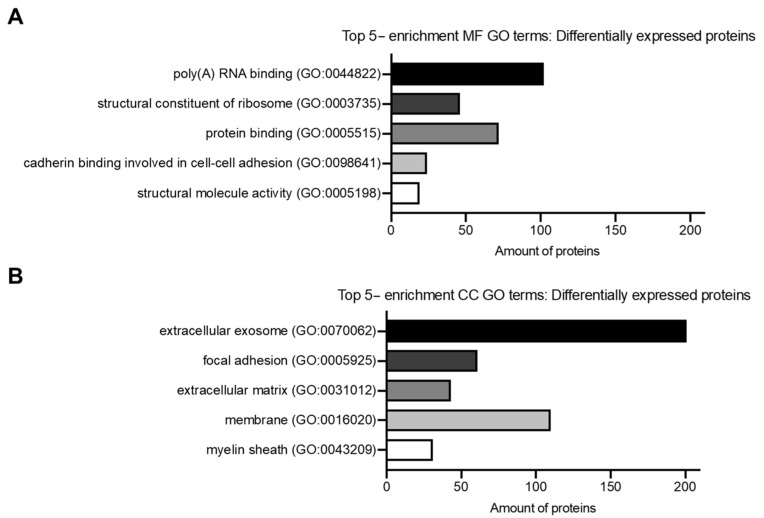
GO Enrichment Analysis of DAPs. (**A**) Top 5- differentially regulated proteins for enrichment GO MF terms, with the number of proteins on each enrichment GO term. (**B**) Top 5 differentially regulated proteins for enrichment GO CC terms, with the number of proteins on each enrichment GO term.

The top enriched GO terms for CC were: “extracellular exosome (GO:0070062)” with 201 proteins, “focal adhesion (GO:0005925)” with 61, “extracellular matrix (GO:0031012)” with 43, “membrane (GO:0016020)” with 110, and “myelin sheath (GO:0043209)” with 31. For MF, the most enriched terms were: “poly(A) RNA binding (GO:0044822)” with 102 proteins, “structural constituent of ribosome (GO:0003735)” with 46, “protein binding (GO:0005515)” with 72, “cadherin binding involved in cell–cell adhesion (GO:0098641)” with 24, and “structural molecule activity (GO:0005198)” with 19. Figure 3A,B summarizes the top five enriched GO terms among differentially abundant proteins.

### 3.3. Biological Characterization of OTM Exclusive Proteins

**Figure 4:**
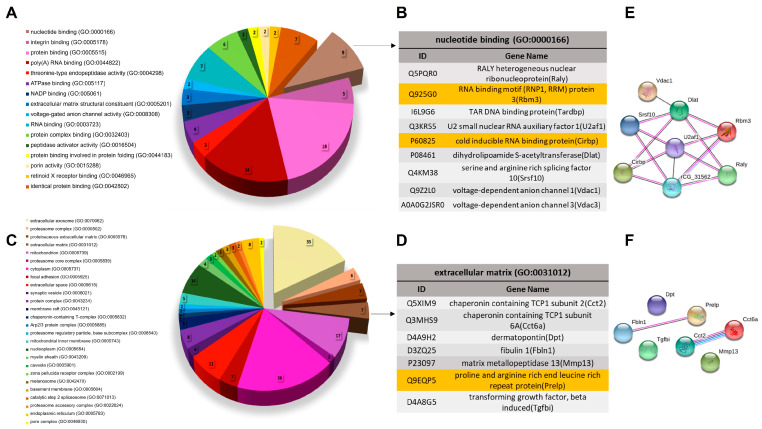
GO Enrichment Analysis of exclusive proteins of OTM. DAVID platform generated the results based on MF and CC. (**A**) Group OTM GO MF terms. The only GO MF term enrichment was “nucleotide binding (GO:0000166)”. (**B**) Group M GO CC terms. The GO CC enrichment terms were “proteinaceous extracellular matrix (GO:0005578)”, “extracellular exosome (GO:0070062)”, “proteasome complex (GO:0000502)” and “extracellular matrix (GO:0031012)”. (**C**) Proteins presented on GO MF enrichment term “nucleotide binding (GO:0000166)”. The proteins in yellow are the chosen ones for immunohistochemistry analysis (IHC). (**D**) Proteins presented on GO CC enrichment term “extracellular matrix (GO:0031012)”. The proteins in yellow are the chosen ones for IHC. (**E**) Low-confidence (0.150) protein–protein interaction network analysis of proteins of GO MF enrichment term “nucleotide binding (GO:0000166)”. (**F**) Low-confidence (0.150) protein–protein interaction network analysis of proteins of GO CC enrichment term “extracellular matrix (GO:0031012)”.

Biological characterization of proteins exclusively identified in the OTM group revealed that the most enriched GO MF term was “nucleotide binding (GO:0000166),” encompassing nine proteins, including Rbm3 and Cirbp. For GO CC terms, the top enrichments were “extracellular exosome (GO:0070062)” with 35 proteins, “proteasome complex (GO:0000502)” with six proteins, and both “proteinaceous extracellular matrix (GO:0005578)” and “extracellular matrix (GO:0031012)” with seven proteins each, the latter including Prelp (Figure 4A–D).

Protein–protein interaction analysis using the STRING platform was conducted for the enriched GO terms “nucleotide binding” and “extracellular matrix,” using a low-confidence interaction score (0.150). Proteins associated with “nucleotide binding” exhibited a densely connected network, indicating strong interactions among them (Figure 4E). Within the “extracellular matrix” category, a notable interaction was observed among Prelp, Fbln1 (fibulin-1), and the chaperonins Cct2 and Cct6a (Figure 4F). These interactions provide insight into potential functional cooperation and molecular mechanisms underlying OTM-induced proteomic changes.

### 3.4. Subcellular Localization and Abundance Pattern of Selected Proteins Determined by IHC Analysis

**Figure 5:**
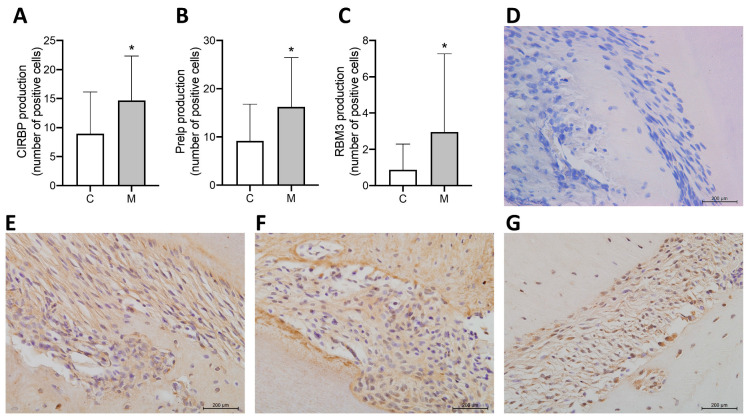
IHC in situ validation of proteins. (**A**–**C**) Mean and standard deviation of the number of positive cells for Cirbp, Prelp, and Rbm3. * Statistical significance (*p* < 0.05). (**D**–**G**) Representative images (400× magnification) of IHC staining for negative control and their respective proteins (Cirbp, Prelp, and Rbm3).

The proteins prolargin (Prelp), cold-inducible RNA-binding protein (Cirbp), and RNA-binding motif protein 3 (Rbm3), identified through proteomic analysis, were validated by IHC staining in periodontal tissues. IHC revealed a significantly higher number of positively stained cells in the OTM group compared to the control group for all three proteins, indicating increased abundance in the PDL in response to mechanical force. Staining was predominantly observed in fibroblast-like cells within the PDL, especially in areas adjacent to alveolar bone and cementum surfaces (Figure 5).

## 4. Discussion

Proteomic analysis of PDL tissues subjected to OTM provides critical insights into the molecular mechanisms underlying this complex biological process. This study successfully identified a robust proteomic profile from laser microdissected PDL samples, demonstrating the sensitivity and effectiveness of this approach for proteomic research. Importantly, the identification of Prelp, Cirbp, and Rbm3, which have not been previously reported in proteomic studies of OTM, highlights the originality of the findings and points to their potential roles in periodontal remodeling under mechanical stress. While not intended for direct clinical translation, this model allows for controlled analysis of protein changes during OTM.

Previous study by Zhang et al. [25] characterized plasma protein profiles from patients undergoing controlled mechanical stress during OTM, identifying 16 differentially abundant proteins, including serotransferrin, fibronectin, and galectin-3-binding protein. These proteins were primarily associated with inflammatory and vesicle-mediated transport processes. Particularly, galectin-3-binding protein abundance was validated using human periodontal ligament (hPDL) cells under mechanical loading, reinforcing its role as a force-responsive molecule 22. This underscores the significance of inflammatory and stress-responsive pathways in orthodontic tooth movement.

Additional studies have focused on saliva proteome alterations in orthodontic contexts, including OTM [26,27], orthodontically induced inflammatory root resorption [28], and accelerated osteogenic orthodontic interventions [29]. Ellias, Zainal Ariffin, Karsani, Abdul Rahman, Senafi and Megat Abdul Wahab [26] identified differential abundance of inflammation-related proteins such as S100-A9, immunoglobulin J chain, Ig alpha-1 chain C region, and CRISP-3, reflecting the activation of local inflammatory and resorptive responses during OTM. Furthermore, Zhang et al. [30] demonstrated distinct salivary proteomic signatures between periodontitis patients and healthy controls undergoing OTM, suggesting that periodontal health significantly influences the proteomic response to mechanical loading.

In the present investigation, IHC analysis validated the upregulation of three selected proteins—Prelp, Rbm3, and Cirbp—in periodontal tissues subjected to mechanical stress. Prelp (proline/arginine-rich end leucine-rich repeat protein) is a small leucine-rich proteoglycan crucial for maintaining connective tissue integrity by anchoring basement membranes and modulating extracellular matrix (ECM) organization [31]. It possesses a unique N-terminal domain enriched with proline and positively charged amino acids, facilitating interactions with chondrocytes and inhibiting osteoclast differentiation [32]. Prelp’s capability to influence stem cell differentiation and support ligament regeneration has been documented previously [33]. Elevated Prelp levels in rheumatoid arthritis have also been interpreted as adaptive responses aimed at limiting osteoclastogenesis and reducing tissue degradation [34]. Thus, our finding of Prelp’s increased presence in mechanically stressed PDL tissue indicates its potential protective and regulatory role in periodontal remodeling during orthodontic treatment.

Rbm3 and Cirbp belong to a family of cold-inducible RNA-binding proteins that facilitate cellular adaptation during stress conditions by enhancing translation and protecting mRNA stability. Their selective abundance in mechanically stressed periodontal tissues strongly suggests involvement in cellular stress response and tissue homeostasis under orthodontic forces. Rbm3 plays a multifaceted role, contributing to skeletal muscle preservation [35], neuroprotection, anti-apoptotic effects, and cell proliferation [36]. Similarly, Cirbp has been implicated in responses to hypoxia and systemic inflammation, notably during sepsis, where it mediates immune and endothelial activation [37,38,39]. Recent studies also indicate Cirbp’s potential as a therapeutic target in inflammatory diseases due to its ability to modulate neutrophil extracellular traps (NETs) and inducible nitric oxide synthase (iNOS) expression [40].

The combined abundance of Prelp, Rbm3, and Cirbp in periodontal tissues under orthodontic forces underscores their collective involvement in tissue remodeling and adaptive cellular responses [35,37,38,39]. These proteins likely collaborate to maintain periodontal tissue integrity, modulate inflammation, and facilitate efficient tissue adaptation and regeneration in response to mechanical stress [30,34]. Nevertheless, the exact mechanistic contributions of these proteins during orthodontic tooth movement remain to be further elucidated through targeted functional studies.

Thant et al. [41] also utilized laser capture microdissection to perform an extracellular matrix–oriented proteomic analysis of the PDL under orthodontic force. On the compression side, they observed upregulation of proteases, matrix metalloproteinases, and annexins, suggesting active matrix degradation and tissue remodeling. Conversely, on the tension side, there was a decrease in mineralization-related proteins and an increase in collagen-modifying enzymes. The methodology used in their study closely aligns with that of the present work, underscoring the sensitivity and precision of laser microdissection for proteomic analysis of periodontal tissues under mechanical stress.

This study provides novel insights into the molecular mechanisms of orthodontically induced periodontal remodeling, identifying Prelp, Rbm3, and Cirbp as proteins with potential regulatory roles. Despite these contributions, several limitations warrant consideration. The proteomic analysis employed a bottom-up, label-free quantification strategy which, although robust, lacks the resolution to distinguish intact proteoforms and post-translational modifications, and may underdetect low-abundance proteins, potentially overlooking biologically relevant variants [42]. Moreover, while the periodontal ligament was precisely isolated via laser microdissection, regional variations (e.g., mesial vs. distal sites) were not independently assessed, limiting spatial specificity. Additionally, as this study focused on a single timepoint (15 days), it does not capture the temporal dynamics of protein expression throughout the course of OTM. Although selected targets were validated through immunohistochemistry, further functional analyses are required to elucidate their roles in periodontal adaptation to mechanical forces. Future studies integrating top-down or orthogonal proteomic approaches will be essential to comprehensively address proteoform-level complexity.

## 5. Conclusions

This study identified distinct proteomic profiles in periodontal ligament tissues during orthodontic tooth movement. Significant changes in protein abundance were observed, with Prelp, Rbm3, and Cirbp emerging as proteins potentially involved in periodontal remodeling. These findings contribute to a deeper understanding of the molecular mechanisms underlying orthodontically induced tissue adaptation.

## Figures and Tables

**Table 1:** Top 10 DAPs in Control and OTM groups. Beta-binomial statistical test applied to NSC values, with significance defined by a log_2_ fold change > 1.5 and a −log_10_(*p*-value) ≥ 1.

Protein Symbol	Description	Fold-Change
Upregulated in OTM		
Prelp	Prolargin	7,867,141.88
Downregulated in OTM		
D3zn79	Similar to 60S ribosomal protein L35	−36,595,282.2
S10a4	Protein S100-A4	−34,574,876.8
D3zku5	Similar to ribosomal protein L31	−20,157,790.6
Q9wuh9	Fibrillin-2	−18,039,348.1
Aqp1	Aquaporin-1	−15,943,980
Crip1	Cysteine-rich protein 1	−14,228,021.1
D3zjd3	Similar to ribosomal protein L28	−11,804,227.7
Hmgb2	High mobility group protein B2	−10,598,886
A0a0h2uhg7	40S ribosomal protein S20	−10,152,529
Imb1	Importin subunit beta-1	−9,620,749.68

## Data Availability

The data supporting the findings of this study are openly available in the PRIDE repository under the following project identifiers: PXD055817 and PXD033647. These datasets are part of a broader experimental study involving additional groups and interventions, of which the current manuscript presents a focused analysis on a specific subset of experimental conditions.

## Supplementary Materials

The following supporting information can be downloaded at: https://www.mdpi.com/article/10.3390/proteomes13030042/s1, Table S1: DAPs, Table S2: Proteomic data of Control group, Table S3: Proteomic data of OTM group.
