# Supplementary material for: Proteomic Analysis of the Periodontal Ligament During Orthodontic Movement: A Study in Rats
Source: Proteomes. 2025 Sep 11;13(3):42. doi: 10.3390/proteomes13030042 (PMC12452640; doi:10.3390/proteomes13030042)
Supplement: Supplementary file 1 [file proteomes-13-00042-s001.zip › Table S1.pdf]

Supplementary Material 1 - Proteomic Analysis of the Periodontal Ligament During Orthodontic Movement: A Study in Rats

Summary table 1. DAPs.

Accession	Description	Protein Symbol	Test-T	Fold-Change
Up-regulated in orthodontics				
Q9EQP5	Prolargin	PRELP	0.00975972	7867141.88
Down-regulated in orthodontics				
A0A0G2K013	Alpha-actinin-4	A0A0G2K013	0.03787926	-1.61409258
F1M779	Clathrin heavy chain	F1M779	0.03162586	-1.76532938
Q5XFX0	Transgelin-2	TAGL2	0.01025367	-1.78629547
Q5FWY9	Col4a1 protein (Fragment)	Q5FWY9	0.02397958	-1.87425275
Q6GMN8	Actn1 protein	Q6GMN8	0.00482586	-1.97862704
Q811A3	Procollagen-lysine,2-oxoglutarate 5-dioxygenase 2	PLOD2	0.01792232	-1.99761887
Q9QX80	CArG-binding factor A	Q9QX80	0.03332196	-2.01585608
P02680	Fibrinogen gamma chain	FIBG	0.00941939	-2.04583022
G3V9I0	Procollagen-lysine,2-oxoglutarate 5-dioxygenase 2	G3V9I0	0.01733335	-2.05579327
Q80ZA3	Alpha-2 antiplasmin	Q80ZA3	0.02187836	-2.05922116
Q6P6G9	Heterogeneous nuclear ribonucleoprotein A1	Q6P6G9	0.00723853	-2.07636987
A0A0G2JSH9	Peroxiredoxin-2	A0A0G2JSH9	0.03151775	-2.10392917
P50123	Glutamyl aminopeptidase	AMPE	0.00126608	-2.11363314
A0A0G2JTI8	Aminopeptidase	A0A0G2JTI8	0.00187376	-2.13233807
P20059	Hemopexin	HEMO	0.02953439	-2.25993728
Q63555	SP120	Q63555	0.02968848	-2.26844769
Q6S3A0	Plectin	Q6S3A0	0.018838	-2.27205293
P30427	Plectin	PLEC	0.018838	-2.27205293
Q5U300	Ubiquitin-like modifier-activating enzyme 1	UBA1	0.04068104	-2.3055252
P85108	Tubulin beta-2A chain	TBB2A	0.0157494	-2.35866974
P38983	40S ribosomal protein SA	RSSA	0.01121418	-2.38815919
G3V7C6	Tubulin beta chain	G3V7C6	0.01744879	-2.38829412
D4A6W6	Similar to 60S ribosomal protein L8	D4A6W6	0.02086149	-2.41016635
P15999	ATP synthase subunit alpha, mitochondrial	ATPA	0.02030251	-2.41568401
O35142	Coatomer subunit beta'	COPB2	0.02424905	-2.41854708
Q3KRF2	High density lipoprotein binding protein (Vigilin)	Q3KRF2	0.01728452	-2.41882489
A0A1W2Q6E9	Moesin	A0A1W2Q6E9	0.02028027	-2.43254928
G3V826	Transketolase	G3V826	0.03458156	-2.433024
G3V928	LDL receptor-related protein 1	G3V928	0.02440544	-2.44480933

O55215	Ribosomal protein S2	O55215	0.01086466	-2.45533035
P50137	Transketolase	TKT	0.03425582	-2.45757083
F1LTJ5	Uncharacterized protein	F1LTJ5	0.04694722	-2.54025462
Q01129	Decorin	PGS2	0.01402027	-2.54132228
Q63083	Nucleobindin-1	NUCB1	0.02855641	-2.62019296
A0A0G2K2V6	Keratin, type I cytoskeletal 10	A0A0G2K2V6	0.01314573	-2.63425533
P85973	Purine nucleoside phosphorylase	PNPH	0.01002174	-2.67541529
P69897	Tubulin beta-5 chain	TBB5	0.00698178	-2.67886303
P19945	60S acidic ribosomal protein P0	RLA0	0.04783001	-2.69532302
Q4AEF8	Coatomer subunit gamma-1	COPG1	0.00632006	-2.6958038
D3ZLL8	40S ribosomal protein S15a-like	D3ZLL8	0.04391238	-2.72062146
A0A096MKE0	Aspartate-beta-hydroxylase	A0A096MKE0	0.04770308	-2.72756757
P45592	Cofilin-1	COF1	0.0053796	-2.73067832
P19944	60S acidic ribosomal protein P1	RLA1	0.01882892	-2.74600854
Q6P7A7	Dolichyl-diphosphooligosaccharide--protein glycosyltransferase subunit 1	Q6P7A7	0.00648434	-2.764056
Q66X93	Staphylococcal nuclease domain-containing protein 1	SND1	0.00323757	-2.81804936
P04636	Malate dehydrogenase, mitochondrial	MDHM	0.01635927	-2.8765152
P11598	Protein disulfide-isomerase A3	PDIA3	0.00267281	-2.90352188
Q4KM66	LOC500183 protein	Q4KM66	0.0166716	-2.93407038
A0A0G2K757	Dolichyl-diphosphooligosaccharide--protein glycosyltransferase subunit 2	A0A0G2K757	0.03293367	-2.99077441
A1L114	Fga protein	A1L114	0.01724845	-3.06662681
P62260	14-3-3 protein epsilon	1433E	0.00887326	-3.10297614
P63102	14-3-3 protein zeta/delta	1433Z	0.01206401	-3.11205237
Q9EPB1	Dipeptidyl peptidase 2	DPP2	0.00547942	-3.13143468
D4ACB8	Chaperonin subunit 8 (Theta) (Predicted), isoform CRA_a	D4ACB8	0.01576712	-3.18967421
Q7M0E3	Destrin	DEST	0.00921165	-3.19146822
Q9JJ54	Heterogeneous nuclear ribonucleoprotein D0	HNRPD	0.04980863	-3.21786802
Q4KM73	UMP-CMP kinase	KCY	0.03732068	-3.35451107
Q9ESN0	Protein Niban	NIBAN	0.02455037	-3.35471618
P47853	Biglycan	PGS1	0.01143733	-3.35879948
P61983	14-3-3 protein gamma	1433G	0.02543993	-3.36278617
Q4QQV0	Tubulin beta chain	Q4QQV0	0.00269066	-3.3684899
Q5RKI0	WD repeat-containing protein 1	WDR1	0.03528301	-3.37095602
A0A0G2JSH5	Serum albumin	A0A0G2JSH5	0.01232742	-3.39455327
P02770	Serum albumin	ALBU	0.01232742	-3.39455327
Q68FR9	Elongation factor 1-delta	EF1D	0.01664893	-3.40984526

Q06647	ATP synthase subunit O, mitochondrial	ATPO	0.0327635	-3.41999679
O70371	Annexin (Fragment)	O70371	0.00232957	-3.43121507
Q66HH8	Annexin	Q66HH8	0.00232957	-3.43121507
M0R5J4	Uncharacterized protein	M0R5J4	0.00362267	-3.51112065
P60711	Actin, cytoplasmic 1	ACTB	0.03763846	-3.51402352
P62963	Profilin-1	PROF1	0.00388326	-3.51715594
P62718	60S ribosomal protein L18a	RL18A	0.04232548	-3.5542959
M0R757	Elongation factor 1-alpha	M0R757	0.00153353	-3.55985405
A0A140TAF0	RCG62531, isoform CRA_g	A0A140TAF0	0.02416966	-3.56413512
B0K031	60S ribosomal protein L7	B0K031	0.01827462	-3.57466053
A0A0G2JY31	Alpha-1-antiproteinase	A0A0G2JY31	0.01461068	-3.57484185
P09495	Tropomyosin alpha-4 chain	TPM4	0.00666233	-3.73172672
P09895	60S ribosomal protein L5	RL5	0.03172202	-3.82319939
P02466	Collagen alpha-2(I) chain	CO1A2	0.0280653	-3.82445947
A0A0H2UHI5	Serine protease inhibitor	A0A0H2UHI5	0.01542289	-3.82979935
Q5FVG5	Similar to tropomyosin 1, embryonic fibroblast-rat, isoform CRA_c	Q5FVG5	0.00541027	-3.85890139
Q6IRK9	Carboxypeptidase Q	CBPQ	0.01351322	-3.87676628
P11232	Thioredoxin	THIO	0.01852413	-3.91419917
P23514	Coatomer subunit beta	COPB	0.02201574	-3.93340378
A0A0G2JX64	Tropomyosin 1, alpha, isoform CRA_i	A0A0G2JX64	0.00594493	-3.93526049
F1LQ00	Collagen type V alpha 2 chain	F1LQ00	0.02513889	-3.93630937
Q9EQX9	Ubiquitin-conjugating enzyme E2 N	UBE2N	0.01268881	-3.94892549
D3ZE00	Uncharacterized protein	D3ZE00	0.03628355	-3.97852272
Q6P6V0	Glucose-6-phosphate isomerase	G6PI	0.01189902	-3.98462059
Q5RJR9	Serine (Or cysteine) proteinase inhibitor, clade H, member 1, isoform CRA_b	Q5RJR9	0.00186419	-4.00548245
P29457	Serpin H1	SERPH	0.00186419	-4.00548245
M0R4B6	RCG64424	M0R4B6	0.01050222	-4.00586555
P04797	Glyceraldehyde-3-phosphate dehydrogenase	G3P	0.04544419	-4.006756
Q01177	Plasminogen	PLMN	0.02042088	-4.00866884
Q6AZ25	Tropomyosin 1, alpha	Q6AZ25	0.00594491	-4.01389838
A0A0G2JSQ4	Tropomyosin 1, alpha, isoform CRA_p	A0A0G2JSQ4	0.00594491	-4.01389838
P68255	14-3-3 protein theta	1433T	0.01828068	-4.02935832
P08289	Alkaline phosphatase, tissue-nonspecific isozyme	PPBT	0.00157546	-4.06056084
A0A0H2UHM3	Haptoglobin	A0A0H2UHM3	0.01613074	-4.06406891
Q9Z0V5	Peroxiredoxin-4	PRDX4	0.01604876	-4.07542996
D3ZH41	Cytoskeleton-associated protein 4	D3ZH41	0.00219303	-4.08939923

Q5I0E7	Transmembrane emp24 domain-containing protein 9	TMED9	0.03033582	-4.08996591
D4ACN7	Myoferlin	D4ACN7	0.0164031	-4.12561054
P62961	Nuclease-sensitive element-binding protein 1	YBOX1	0.03932618	-4.1285077
M0R907	RCG60635, isoform CRA_b	M0R907	0.0183911	-4.16390938
A0A0G2K531	Glutathione peroxidase	A0A0G2K531	0.02145963	-4.17360761
P10111	Peptidyl-prolyl cis-trans isomerase A	PPIA	0.00330876	-4.18430205
D3ZRN3	Actin, beta-like 2	D3ZRN3	0.00564136	-4.24262166
P51886	Lumican	LUM	0.00254102	-4.25025596
F1LNF1	Heterogeneous nuclear ribonucleoproteins A2/B1	F1LNF1	0.00022758	-4.29656725
G3V9Y1	Myosin, heavy polypeptide 10, non-muscle, isoform CRA_b	G3V9Y1	0.02056292	-4.3062605
A0A0G2K4M6	Actin, aortic smooth muscle	A0A0G2K4M6	0.01159757	-4.32014744
D4A6A2	Heterogeneous nuclear ribonucleoprotein A3	D4A6A2	0.00642951	-4.39644102
G3V763	Collagen alpha-1(V) chain	G3V763	0.02398339	-4.41203304
Q63610	Tropomyosin alpha-3 chain	TPM3	0.00617663	-4.41756481
B0BNI5	Olfactomedin-like protein 3	OLFL3	0.01450314	-4.42137724
P13471	40S ribosomal protein S14	RS14	0.01545643	-4.42349601
P85845	Fascin	FSCN1	0.00028375	-4.4291601
Q5M842	IgG-2a protein	Q5M842	0.00634049	-4.49990584
P02650	Apolipoprotein E	APOE	0.01873884	-4.50649115
Q6P3V8	Eukaryotic translation initiation factor 4A1	Q6P3V8	0.02337259	-4.53483977
P18418	Calreticulin	CALR	0.00157703	-4.54507785
Q4V8I6	60S ribosomal protein L11	Q4V8I6	0.01101474	-4.55013658
P14841	Cystatin-C	CYTC	0.00991211	-4.55172104
A0A0G2K8B7	Eukaryotic initiation factor 4A-II	A0A0G2K8B7	0.00943245	-4.64397321
Q3MIE4	Synaptic vesicle membrane protein VAT-1 homolog	VAT1	0.00567894	-4.64565874
Q4KLJ1	RCG61762, isoform CRA_a	Q4KLJ1	0.04414583	-4.65578647
A0A0G2K8K0	Splicing factor proline and glutamine rich	A0A0G2K8K0	0.0236434	-4.69494672
D4A8D5	Filamin B	D4A8D5	0.03662277	-4.70101959
P62250	40S ribosomal protein S16	RS16	0.01965882	-4.70745777
Q9Z1X1	Extended synaptotagmin-1	ESYT1	0.01917639	-4.76955076
A0A0G2K7W6	Similar to 60S ribosomal protein L27a	A0A0G2K7W6	0.0076369	-4.81445549
Q0QEW8	Ribosomal protein L18 (Fragment)	Q0QEW8	0.01285885	-4.84420314
P61314	60S ribosomal protein L15	RL15	0.00695113	-4.90232634
Q5U362	Annexin	Q5U362	0.00410624	-4.90319233
P05065	Fructose-bisphosphate aldolase A	ALDOA	0.00232628	-4.92723488

A0A0G2JUA5	AHNAK nucleoprotein	A0A0G2JUA5	0.00406549	-4.94125994
Q6AYC4	Macrophage-capping protein	CAPG	0.02280171	-4.95720507
F1M013	60S ribosomal protein L7a	F1M013	0.00508307	-4.99748954
D4A6G6	Ribosomal protein S19-like	D4A6G6	0.03069469	-4.99874896
X1WI37	40S ribosomal protein S4	X1WI37	0.00632543	-5.02782109
A0A0G2JWX4	Keratin, type II cytoskeletal 2 epidermal	A0A0G2JWX4	0.01102781	-5.05746983
Q6P790	60S ribosomal protein L6 (Fragment)	Q6P790	0.01075641	-5.07053462
F1M853	Ribosome-binding protein 1	F1M853	0.00272301	-5.15214093
P70490	Lactadherin	MFGM	0.0377879	-5.15540804
Q794E4	Heterogeneous nuclear ribonucleoprotein F	HNRPF	0.00052587	-5.22207851
A0A0G2K3Z9	Uncharacterized protein	A0A0G2K3Z9	0.00513896	-5.22776632
P06761	78 kDa glucose-regulated protein	GRP78	0.00381762	-5.28364312
A0A0G2JYF7	Catenin alpha 1	A0A0G2JYF7	0.01970497	-5.29424947
A0A0G2K9Y0	Immunoglobulin heavy constant mu	A0A0G2K9Y0	0.01289889	-5.29754069
P62198	26S proteasome regulatory subunit 8	PRS8	0.01592988	-5.31865216
D4A2G6	Thrombospondin 2	D4A2G6	0.01793187	-5.38108369
P35427	60S ribosomal protein L13a	RL13A	0.00495627	-5.38985547
Q6PDV8	RCG31311	Q6PDV8	0.01366181	-5.40394536
P04897	Guanine nucleotide-binding protein G(i) subunit alpha-2	GNAI2	0.00997746	-5.40629725
A9UMV8	Histone H2A.J	H2AJ	0.0011837	-5.40792639
D4ACV3	Histone H2A	D4ACV3	0.00118199	-5.43948581
P63018	Heat shock cognate 71 kDa protein	HSP7C	0.00352477	-5.4459345
F1LSW7	60S ribosomal protein L14	F1LSW7	0.00343381	-5.5466334
Q6LDS4	Superoxide dismutase	Cu-Zn] OS=Rattus norvegicus GN=Sod1 PE=2 SV=1 -	0.02964866	-5.55595337
D3ZZN4	Uncharacterized protein	D3ZZN4	0.01785287	-5.59969212
F1LPK7	Plastin 3 (T-isoform), isoform CRA_a	F1LPK7	0.00689356	-5.63120495
Q68FP1	Gelsolin	GELS	0.00054298	-5.78534349
F1LN88	Aldehyde dehydrogenase, mitochondrial	F1LN88	0.00390805	-5.79437454
G3V9R9	Afamin	G3V9R9	0.03326097	-5.86777896
P29314	40S ribosomal protein S9	RS9	0.00380526	-5.89527143
P04906	Glutathione S-transferase P	GSTP1	0.00601194	-5.97255734
G3V843	Prothrombin	G3V843	0.04157763	-6.02201474
A0A0H2UHL3	Adipocyte enhancer-binding protein 1	A0A0H2UHL3	0.00253415	-6.12075953
Q6P3V9	60S ribosomal protein L4	Q6P3V9	0.00033525	-6.22147253
Q05175	Brain acid soluble protein 1	BASP1	0.00606123	-6.23244386

M0RD75	40S ribosomal protein S6	M0RD75	0.03034995	-6.30105738
Q6IFX1	Keratin, type I cytoskeletal 24	K1C24	0.04426281	-6.36708025
Q64240	Protein AMBP	AMBP	0.01022841	-6.39972905
P82995	Heat shock protein HSP 90-alpha	HS90A	0.00176941	-6.40762014
P08494	Matrix Gla protein	MGP	0.02669472	-6.426888
P51635	Alcohol dehydrogenase	NADP(+)] OS=Rattus norvegicus GN=Akr1a1 PE=1 SV=2 -	0.0043253	-6.43134389
P13941	Collagen alpha-1(III) chain	CO3A1	0.00143841	-6.44362493
A0A0G2JXC1	Protein ERGIC-53	A0A0G2JXC1	0.00067042	-6.44959351
F1LMW7	Myristoylated alanine-rich C-kinase substrate	F1LMW7	0.00386664	-6.45766137
P42123	L-lactate dehydrogenase B chain	LDHB	0.03609724	-6.50467245
P34058	Heat shock protein HSP 90-beta	HS90B	0.00133898	-6.63028697
Q10758	Keratin, type II cytoskeletal 8	K2C8	0.00574791	-6.64405217
B2GVB4	Sept9 protein (Fragment)	B2GVB4	0.00188135	-6.65664923
A0A0G2JXC3	40S ribosomal protein S21	A0A0G2JXC3	0.0013976	-6.67991678
D4A4D5	Similar to 60S acidic ribosomal protein P2	D4A4D5	0.004115	-6.70785799
Q6PDU1	Serine/arginine-rich splicing factor 2	SRSF2	0.00083329	-6.77579881
A0A0G2K0X9	Protein transport protein Sec31A	A0A0G2K0X9	0.00117988	-6.78575129
A0A0A0MXW3	Histone H2A	A0A0A0MXW3	0.00195727	-6.80342901
P62804	Histone H4	H4	0.00127025	-6.8063736
P35213	14-3-3 protein beta/alpha	1433B	0.00819411	-6.81536165
O88989	Malate dehydrogenase, cytoplasmic	MDHC	0.00648345	-6.84457178
Q6IFV4	Keratin, type I cytoskeletal 13	K1C13	0.006997	-6.85573819
A0A0G2KA35	Uncharacterized protein	A0A0G2KA35	0.00175347	-6.9008729
D3ZJ08	Histone H3	D3ZJ08	0.00432029	-6.92109675
Q63030	Rat alpha-smooth muscle actin mRNA (Fragment)	Q63030	0.0032003	-6.95756195
D4A5L9	Similar to Cytochrome c, somatic	D4A5L9	0.00341486	-7.04653986
I6L9G5	Rcn3 protein	I6L9G5	0.0012454	-7.06941922
Q5U2Z3	Nucleosome assembly protein 1-like 4	NP1L4	0.00816195	-7.10699391
G3V8L3	Lamin A, isoform CRA_b	G3V8L3	0.0015808	-7.1447385
A0A068F1Y2	Beta-actin (Fragment)	A0A068F1Y2	0.00131121	-7.21410269
Q3B7V5	RAB2B, member RAS oncogene family	Q3B7V5	0.00677098	-7.2374733
A0A0G2K722	Uncharacterized protein	A0A0G2K722	0.01726307	-7.24492661
Q6LED0	Histone H3.1	H31	0.00915674	-7.26012017
D3ZK97	Histone H3	D3ZK97	0.00631369	-7.27453127
M0RAV0	Uncharacterized protein	M0RAV0	0.01273786	-7.36487421

P61354	60S ribosomal protein L27	RL27	0.01139972	-7.40705951
Q5XI34	Protein phosphatase 2 (Formerly 2A), regulatory subunit A (PR 65), alpha isoform, isoform CRA_a	Q5XI34	0.00563965	-7.53270093
P84100	60S ribosomal protein L19	RL19	0.00210606	-7.64671015
D3ZZC1	RCG43947	D3ZZC1	0.00666391	-7.651416
P10760	Adenosylhomocysteinase	SAHH	0.00211501	-7.67651887
D3ZBN0	Histone H1.5	H15	0.00084702	-7.73279957
Q62636	Ras-related protein Rap-1b	RAP1B	0.00146323	-7.78032256
A1L1J8	RAB5B, member RAS oncogene family	A1L1J8	0.03699191	-7.94848261
F1LQ14	60S ribosomal protein L34	F1LQ14	0.00323526	-7.96937173
D3ZRM9	60S ribosomal protein L13	D3ZRM9	0.00345849	-7.97472271
Q9Z1B2	Glutathione S-transferase Mu 5	GSTM5	0.00614072	-8.10820807
Q5XI32	F-actin-capping protein subunit beta	CAPZB	0.00062102	-8.15037772
A0A0G2K3A6	Ighg protein-like	A0A0G2K3A6	0.00656593	-8.16421996
A0JN30	Canopy 2 homolog (Zebrafish)	A0JN30	0.00147049	-8.18972179
A0A0G2JYA4	Serine/threonine-protein phosphatase	A0A0G2JYA4	0.00928423	-8.22419382
G3V9E3	Caldesmon 1, isoform CRA_b	G3V9E3	0.00503629	-8.26240643
P06685	Sodium/potassium-transporting ATPase subunit alpha-1	AT1A1	0.00197151	-8.27127795
Q5U2V1	Peptidylprolyl isomerase	Q5U2V1	0.00121692	-8.32551392
A0A0G2JYL4	Prolyl 4-hydroxylase subunit alpha 2	A0A0G2JYL4	0.00185878	-8.37674864
P05544	Serine protease inhibitor A3L	SPA3L	0.00413765	-8.3771073
P61805	Dolichyl-diphosphooligosaccharide--protein glycosyltransferase subunit DAD1	DAD1	0.00084238	-8.46379284
A0A0G2K290	Uncharacterized protein	A0A0G2K290	0.00616621	-8.46958193
D3ZWE0	Histone H2A	D3ZWE0	0.00074062	-8.52743164
Q6AY25	Transmembrane emp24 domain-containing protein 3	TMED3	0.00142971	-8.54970686
G3V9M6	Fibrillin 1	G3V9M6	0.0058929	-8.564155
A0A0G2K654	Histone cluster 1 H1 family member c	A0A0G2K654	0.00058088	-8.58946459
Q6IFV1	Keratin, type I cytoskeletal 14	K1C14	0.0065993	-8.62113595
P05964	Protein S100-A6	S10A6	0.00518382	-8.66075583
A0A0G2JTG7	Heterogeneous nuclear ribonucleoprotein H	A0A0G2JTG7	0.00119035	-8.70672804
P84586	RNA-binding motif protein, X chromosome retrogene-like	RMXRL	0.00581601	-8.71417191
P31000	Vimentin	VIME	0.00010544	-8.86830954
D3ZJW6	RCG21066	D3ZJW6	0.00871295	-8.90869218
P10860	Glutamate dehydrogenase 1, mitochondrial	DHE3	0.03077284	-9.05535083

P81155	Voltage-dependent anion-selective channel protein 2	VDAC2	0.00914315	-9.08629915
G3V8A5	Vacuolar protein sorting-associated protein 35	G3V8A5	0.00023317	-9.12196195
Q5PPP1	Clathrin light chain	Q5PPP1	0.00030909	-9.12903953
P62890	60S ribosomal protein L30	RL30	0.00146823	-9.25993507
A0A0G2JSL0	Proteasome subunit beta type	A0A0G2JSL0	0.00201269	-9.33848786
A0A0H2UHT6	Ribosomal protein S18	A0A0H2UHT6	0.00046319	-9.36816651
Q62902	Protein ERGIC-53	LMAN1	0.0005178	-9.46543319
P50503	Hsc70-interacting protein	F10A1	0.03021411	-9.62451686
A0A0G2JZS2	Polyadenylate-binding protein 1	A0A0G2JZS2	0.00031752	-9.67605509
Q6IFU9	Keratin 16	Q6IFU9	0.00067465	-9.77635293
P62243	40S ribosomal protein S8	RS8	0.0023362	-9.83225477
B2RZ72	Actin-related protein 2/3 complex subunit 4	B2RZ72	0.00123504	-9.92805823
P43278	Histone H1.0	H10	0.0040267	-10.0091941
G3V8G5	Golgi apparatus protein 1	G3V8G5	0.00582904	-10.014428
Q6AY09	Heterogeneous nuclear ribonucleoprotein H2	HNRH2	5.9232E-05	-10.0990556
Q6P9U8	Eukaryotic translation initiation factor 3 subunit H	EIF3H	0.00447536	-10.3858161
P38659	Protein disulfide-isomerase A4	PDIA4	0.00137662	-10.4016236
Q4V8F6	Pcbp2 protein	Q4V8F6	0.00357939	-10.4382699
P20767	Ig lambda-2 chain C region	LAC2	0.00045257	-10.5218318
A0A140TAA4	Programmed cell death 6-interacting protein	A0A140TAA4	0.0036172	-10.5231102
Q5RJR8	Leucine-rich repeat-containing protein 59	LRC59	0.00501669	-10.7202558
P00173	Cytochrome b5	CYB5	0.00063248	-10.8628723
A0A0G2JSK5	Integrin beta	A0A0G2JSK5	0.01084014	-10.8971292
Q5PQK2	FUS RNA-binding protein	Q5PQK2	0.00041758	-10.9836704
O35244	Peroxiredoxin-6	PRDX6	0.00130712	-11.0924097
Q66HF1	NADH-ubiquinone oxidoreductase 75 kDa subunit, mitochondrial	NDUS1	0.00101147	-11.4628321
A0A0G2JZH3	Thrombospondin 3	A0A0G2JZH3	3.6673E-05	-11.535658
O08628	Procollagen C-endopeptidase enhancer 1	PCOC1	0.00037158	-11.9379744
P62278	40S ribosomal protein S13	RS13	0.01243921	-11.9867139
G3V741	Phosphate carrier protein, mitochondrial	G3V741	0.00315521	-12.0911456
Q6AXS5	Plasminogen activator inhibitor 1 RNA-binding protein	PAIRB	0.00042783	-12.1443582
P14562	Lysosome-associated membrane glycoprotein 1	LAMP1	0.00097093	-12.6431388
M0RA26	Ribosomal protein S27-like	M0RA26	0.0004125	-12.6526039

P81795	Eukaryotic translation initiation factor 2 subunit 3, X-linked	IF2G	0.00181506	-13.1414584
P97586	Cell growth regulator with EF hand domain protein 1	CGRE1	0.00629046	-13.1566443
P47875	Cysteine and glycine-rich protein 1	CSRP1	0.00067493	-13.5228146
F1LV13	Heterogeneous nuclear ribonucleoprotein M	F1LV13	0.00062299	-14.1380943
A0A0G2JZS9	Uncharacterized protein	A0A0G2JZS9	0.00105663	-14.2267265
Q4V7D1	Signal sequence receptor, alpha	Q4V7D1	7.8575E-05	-14.6865667
P37361	Metallothionein-3	MT3	0.00491387	-14.9527478
D3ZSY4	Eosinophil peroxidase	D3ZSY4	0.00183313	-14.9794532
P62282	40S ribosomal protein S11	RS11	0.00076951	-15.3110123
G3V7U4	Lamin-B1	G3V7U4	0.00070654	-15.5887354
A0A0H2UHU0	40S ribosomal protein S25	A0A0H2UHU0	0.00337167	-15.6774962
B4F7C9	Serine/threonine-protein kinase Chk1	B4F7C9	0.00023703	-15.9643422
F1M265	Palladin	F1M265	0.00380956	-16.1568445
B5DFB0	Leprecan-like 2 (Predicted), isoform CRA_b	B5DFB0	0.0012926	-16.1630985
Q5XI73	Rho GDP-dissociation inhibitor 1	GDIR1	0.00184246	-16.2002814
P62912	60S ribosomal protein L32	RL32	6.7467E-05	-16.8632503
P40241	CD9 antigen	CD9	0.000696	-17.3793207
Q4KM74	Vesicle-trafficking protein SEC22b	SC22B	0.00010552	-17.4107601
M0RA79	Uncharacterized LOC691828	M0RA79	4.3876E-06	-18.1046219
F1M7X3	Cadherin 13	F1M7X3	9.8235E-06	-19.8282244
G3V9S9	SEC24 homolog D, COPII coat complex component	G3V9S9	0.00039485	-22.1082948
P62832	60S ribosomal protein L23	RL23	0.00048054	-23.0683131
Q63965	Sideroflexin-1	SFXN1	2.2258E-06	-28.3028404
G3V6I9	60S ribosomal protein L26	G3V6I9	0.01862888	-29.4862771
G3V804	Sideroflexin	G3V804	0.03406866	-32.0438413
F1LMS5	Thrombospondin-4	F1LMS5	0.04940533	-34.9950554
D3ZLC1	Lamin B2	D3ZLC1	0.03080185	-41.6179081
Q5RKJ9	RAB10, member RAS oncogene family	Q5RKJ9	0.03202354	-44.5432826
Q5RKG9	Eukaryotic translation initiation factor 4B	Q5RKG9	0.04322657	-1013802.81
F1MA98	Nucleoprotein TPR	TPR	0.04759847	-1875954.7
M0R6K0	Laminin subunit beta-2	M0R6K0	0.03801491	-2121309.2
A0A0G2JUB0	Protein FAM3C	A0A0G2JUB0	0.00846259	-2234653.46
D4ABT8	Heterogeneous nuclear ribonucleoprotein U-like 2	D4ABT8	0.03414933	-2513356.22
F1LW91	Nuclear mitotic apparatus protein 1	F1LW91	0.00544343	-2663326.02

G3V6S1	PRKC apoptosis WT1 regulator protein	G3V6S1	0.04068299	-2666364.12
Q6AZ26	C-terminal binding protein 1	Q6AZ26	0.03161461	-2681637.91
Q60587	Trifunctional enzyme subunit beta, mitochondrial	ECHB	0.03100491	-2720858.49
B0BNA7	Eukaryotic translation initiation factor 3 subunit I	EIF3I	0.03522105	-2956742.58
B5DF03	Peptidylprolyl isomerase (Fragment)	B5DF03	0.04029228	-3088000.18
B2RYQ2	Serine/threonine-protein phosphatase 2A activator	B2RYQ2	0.02155444	-3478012.07
G3V9T1	Cysteine-rich secretory protein LCCL domain-containing 2	G3V9T1	0.037775	-4004527.66
Q6P747	Heterochromatin protein 1-binding protein 3	HP1B3	0.04796221	-4634551.69
Q9Z1H9	Caveolae-associated protein 3	CAVN3	0.03405293	-4669667.69
B2RZ37	Receptor expression-enhancing protein 5	REEP5	0.02577853	-4955588.88
G3V7I0	Peroxiredoxin 3	G3V7I0	0.02318584	-4960045.94
Q5XI78	2-oxoglutarate dehydrogenase, mitochondrial	ODO1	0.04296752	-5217810.66
Q5XI19	Fermitin family homolog 2 (Drosophila)	Q5XI19	0.04385977	-5266361.81
P62329	Thymosin beta-4	TYB4	0.03136708	-5514663.65
A0A0G2JSU3	Protein SET	A0A0G2JSU3	0.00365384	-5651901.45
Q9ES40	PRA1 family protein 3	PRAF3	0.03913386	-5919160.41
D4A0L4	Y-box-binding protein 3	D4A0L4	0.03724882	-6354591.08
P61621	Protein transport protein Sec61 subunit alpha isoform 1	S61A1	0.00960984	-8521119.1
P52296	Importin subunit beta-1	IMB1	0.03161764	-9620749.68
A0A0H2UHG7	40S ribosomal protein S20	A0A0H2UHG7	0.03926698	-10152529
P52925	High mobility group protein B2	HMGB2	0.04881793	-10598886
D3ZJD3	Similar to ribosomal protein L28	D3ZJD3	0.03217364	-11804227.7
P63255	Cysteine-rich protein 1	CRIP1	0.03368212	-14228021.1
P29975	Aquaporin-1	AQP1	0.0471188	-15943980
Q9WUH9	Fibrillin-2	Q9WUH9	0.03878869	-18039348.1
D3ZKU5	Similar to ribosomal protein L31	D3ZKU5	0.03102005	-20157790.6
P05942	Protein S100-A4	S10A4	0.03609191	-34574876.8
D3ZN79	Similar to 60S ribosomal protein L35	D3ZN79	0.04487744	-36595282.2
